# Role of Theories in the Design of Web-Based Person-Centered Support: A Critical Analysis

**DOI:** 10.1155/2014/603047

**Published:** 2014-03-19

**Authors:** Agneta Ranerup, Carina Sparud-Lundin, Ingalill Koinberg, Ingela Skärsäter, Margaretha Jenholt-Nolbris, Marie Berg

**Affiliations:** ^1^Department of applied IT, University of Gothenburg, 412 96 Gothenburg, Sweden; ^2^Centre for Person-Centred Care (GPCC), University of Gothenburg, Box 457, 405 30 Gothenburg, Sweden; ^3^Institute of Health and Care Sciences, Sahlgrenska Academy, University of Gothenburg, Box 457, 405 30 Gothenburg, Sweden; ^4^School of Social and Health Sciences, Halmstad University, Box 823, 301 18 Halmstad, Sweden; ^5^School of Health Sciences, University of Borås, 501 90 Borås, Sweden

## Abstract

*Objective*. The aim of this study was to provide a critical understanding of the role of theories and their compatibility with a person-centered approach in the design and evaluation of web-based support for the management of chronic illness.* Methods*. Exploration of web-based support research projects focusing on four cases: (1) preschool children aged 4–6 with bladder dysfunction and urogenital malformation; (2) young adults aged 16–25 living with mental illness; (3) women with type 1 diabetes who are pregnant or in early motherhood; and (4) women who have undergone surgery for breast cancer. Data comprised interviews with research leaders and documented plans. Analysis was performed by means of a cross-case methodology.* Results*. The used theories concerned design, learning, health and well-being, or transition. All web support products had been developed using a participatory design (PD). Fundamental to the technology design and evaluation of outcomes were theories focusing on learning and on health and well-being. All theories were compatible with a person-centered approach. However, a notable exception was the relatively collective character of PD and Communities of Practice.* Conclusion*. Our results illustrate multifaceted ways for theories to be used in the design and evaluation of web-based support.

## 1. Introduction

Increasingly, different types of web-based support are being developed for patients in managing their illness [1–6]. It has been argued that efficient support for managing chronic illnesses and conditions should be tailored to each person and his or her needs and preferences. In other words, it should be in line with a “person-centered care” philosophy (PCC) [7, 8].

Central to PCC is that the person is highlighted and considered from a broader, holistic perspective than merely in terms of medical status. PCC is based on the patient's experience of his/her situation being a capable and active person with individual conditions and resources and with efforts to preserve their dignity [8–10]. PCC comprises openness and acknowledgement of each person's perspective and special resources which assist them to be self-reflecting and capable of shared decision making despite health-related limitations. PCC brings attention to partnership, including shared power and responsibility between the person and the professional caregiver, with efforts to actively empower and involve the patient in the care process [7].

With this paper we want to highlight the PCC perspective [7] in web-based technology as utilized in the everyday life of persons with long-term illnesses. In a PCC web context there are elements of both prevention and learning for the person with illness and for significant others such as health care professionals, peers, close friends, and relatives.

Designing web-based support compatible with the concept of person-centeredness in this context may start with a foundation of implicit ideas including user views [11–13] and also of theories, for example, [14, 15] which are utilized and affect different parts of the design process. A variety of definitions of “theory” exist. Some definitions have a focus on causality, emphasizing the role of interrelated constructs or concepts representing a systematic view of phenomena, while they simultaneously express a causal relationship between them [16]. In this study, we opt for a broader definition, in accordance with Hall and Schmid Mast [17]. We denote theories as all frameworks, models, and similar including sets of conceptual constructs that explain and describe a phenomenon at a conceptual level [17]. We argue that the use of theories in the design of web-support can be a way of being more explicit about the rationale for its design. Theories can also serve as visible and discussable frameworks for certain web-based care actions.

Previous research on design and use of internet technologies to support patients have featured the process, including requirement generation, design, and pilot testing, as well as evaluations of the outcome of use [18]. Suggested ideals in the design process have also been described [2].

Studies of the role of theories have appeared in the form of original qualitative case studies [14, 15, 18], literature reviews [4, 19], theoretical testing, discussion of the potential to utilize selected theories [15, 20, 21], and in principal views or analyses of theory use [17]. There is, however, a gap of knowledge about the roles of theories in the design and evaluation of web-based technologies to be used by people living with chronic illnesses.

The aim of this study was to provide a critical understanding of the role of theories and their compatibility with a person-centered approach in the design and evaluation of web-based support for the management of chronic illness. The research questions were as follows.

- What types of theories were used?
- How were the theories used?
- To what extent are these theories compatible with a person-centered approach?

In our critical endeavor to expand this knowledge, we explored four different web-based support research projects, here labeled “cases.” The cases represent a variation of different chronic illnesses and different age groups (Table 1).

## 2. Materials and Methods

### 2.1. The Cases

Each case focused on a specific target group with long-term illnesses and included a research intervention including web-based support aiming to facilitate learning and well-being (Table 1). The cases involved persons of different ages: (1) preschool children aged 4–6 with bladder dysfunction and urogenital malformation; (2) young adults (aged 16–25) living with mental illness; (3) women with type 1 diabetes who are pregnant or in early motherhood up to infancy of 6 months; and (4) women who have undergone surgery for breast cancer. Cases 1 and 3 are still ongoing interventions, while Cases 2 and 4 are complete and implemented in ordinary healthcare routines.

Although the cases focused on different target groups with different illnesses or long-term conditions, there were also similarities. All the cases were intervention studies, and representatives from the target group were involved in the design process of the interventions. In all cases, the intervention included information about the specific condition (Cases 1–4). To support self-management in daily life, contact was offered with either peers (Cases 2 and 3) or healthcare professionals (Cases 1 and 2). For more details on each case, please see publications [1, 5, 6, 22].

### 2.2. Procedure for Data Collection and Analysis of Use of Theories

Data was generated in several ways. First, in 2011, the project members/leaders of each case (Carina Sparud-Lundin, Ingalill Koinberg, Ingela Skärsäter, Margaretha Jenholt-Nolbris, and Marie Berg) were interviewed by a member of the research group (not author of this paper). The aim of the interview was to get a broad overview of target groups, the intervention and its aim, and utilized theories of how users' views were collected and which technological devices were used in each case. The interviews lasted 90–120 minutes and notes were taken. Data focusing on user requirements and user participation was also collected. A list of utilized theories was produced. More details are reported elsewhere [22].

As a second step, the first author of this paper (Agneta Ranerup) carried out new interviews in 2012 with the project leaders in Cases  1, 2, and 4 (Ingalill Koinberg, Ingela Skärsäter, and Margaretha Jenholt-Nolbris), and with the project leader and the coleader in Case  3 (Carina Sparud-Lundin and Marie Berg), the focus being specifically on theory use. Questions were asked about the influence and use of theories in association with the basic idea of the project, the organization of the development work, design of the technology, and evaluation of outcomes. The list of theories compiled after the interviews in 2011 was also checked and corrected during the interview. These interviews lasted between 50 and 70 minutes and were recorded and transcribed.

In the third step, all authors (Agneta Ranerup, Carina Sparud-Lundin, Ingalill Koinberg, Ingela Skärsäter, Margaretha Jenholt-Nolbris, and Marie Berg) participated in a stepwise description and analysis of the data. The reason for this was threefold: firstly, to achieve agreement regarding utilized theories in each of the cases (this was particularly important, both for identifying similarities and differences and for creating a mutual understanding), secondly, to generate a relevant understanding of significant aspects of how these theories had been used, and finally to explore connectedness to the concept of person-centeredness.

The analysis was performed by means of a cross-case method that involved the following steps: (1) preparation of a corrected list of theories utilized in the cases. (2) Using this new list as a blueprint, a description of basic aspects of each theory was developed consisting of focus, basic concepts, and important references. These descriptions of particular theories were first prepared by the first author (Agneta Ranerup) and checked and rewritten by all the other authors (Agneta Ranerup, Carina Sparud-Lundin, Ingalill Koinberg, Ingela Skärsäter, Margaretha Jenholt-Nolbris, and Marie Berg). (3) For each theory, its use in the cases was described. (4) Finally, this developed data in terms of theory type and use was analyzed from the perspective of person-centeredness with its core elements described above.

## 3. Results and Discussion

The results are presented in three sections answering the questions: (1) what types of theories were used? (2) How were the theories used? (3) To what extent are these theories compatible with a person-centered approach?

### 3.1. Types of Theories

In total, eight theories were identified as utilized in any of the cases. Using an inductive categorization these theories have been sorted into four categories: (1) design, (2) learning, (3) health and well-being, and (4) transition. An overview of theory use in each case is given in Table 2 and a short description is given below.

#### 3.1.1. Design

*Participatory Design* (PD) is based on the idea that increased involvement of persons affected by a particular change (social or technical) gives a more useful and accepted design outcome [23, 24]. Central aspects of PD therefore concern increased democratization and participation in decision-making for employees in workplaces and capture their true knowledge [24]. The nature of user involvement in PD can vary a great deal. In direct participation, users are deeply involved not only in the design process but also in the decision-making of the project. At the other end of the scale, users are given a more consultative role and the main purpose is to check the design process [24].

#### 3.1.2. Learning

*Communities of Practice.* A perspective on knowing and learning is the concept of Communities of Practice (CoP). CoPs are formed by people sharing common interests. CoP is about knowing, but also about being together, living meaningfully, developing a satisfying identity, and altogether being human. They become integral parts of daily lives and also the one the persons belong to. It is through the process of sharing knowledge and experiences with a CoP that the members may feel community as well as learning and developing. CoPs are now also found in virtual spaces, across a worldwide web of computers. In health care interventions, CoPs can act as a guide to approaching problems, being part of an intervention, and evaluation [25].

*Sociocultural theory* draws heavily on the work of Vygotsky [26]. It focuses on the roles that participation plays in social interactions and culturally organized activities in influencing psychological development. According to Säljö [27], learning occurs in the interaction between individuals, and in a sociocultural perspective, the focus is on how individuals and groups acquire and utilize physical and cognitive resources. From a sociocultural perspective, the computer can be seen as a mediating tool in a knowledge process. Learning can also be seen as self-directed; people can choose content themselves (what they want to take part in or read) and frequency (how often). Thus, the person has increased opportunities to process their own questions.

*Variation theory* is employed as a method for describing learning and how we see and obtain knowledge about the outside world. By describing different aspects of phenomenon, our experiences, including those associated with illness and health, can be understood. The theory also studies relations between individuals on the one hand, and what will be learned on the other, and also describes the variation in perception of different aspects of a phenomenon [28].

#### 3.1.3. Health and Well-Being

*Salutogenic Theory for Health*. A salutogenic perspective is a health promotive perspective. Research shows that people and systems that develop the ability to implement a salutogenic way of living will not only live longer but also perceive they are in good health and enjoy a better quality of life and mental well-being. In addition, they have better than average resilience to stress and more constructive health behaviors. Even if they become ill or get a chronic disease, they will do better than the average [29]. The most well-known and best explored theory with the strongest evidence base is the original Sense of Coherence Theory. It stresses that health and ill-health is not a dichotomy but a continuum in which every person is placed. Furthermore, it focuses on a person's resources in the movement towards health. There are several core concepts in the theory: the Sense of Coherence (SOC), including comprehensibility, manageability, and sense of meaningfulness and the Generalized Resistance Resources (GRRs) [30]. Another way of studying health is to assess a person's self-efficacy, which includes both self-esteem and belief in own ability to handle difficult situations. In addition, observation of how other people succeed in certain situations will increase self-efficacy, especially if the role model has similar conditions. Verbal persuasion means that significant others have an impact through verbalizing the proposed capacity, thus convincing the person that they will manage a situation and causing them to put more energy into doing the job, which, in turn, will make them succeed. The last part comprises physiological and affective states and the impact they have on the person in stressful situations [31].

*Social support* plays a vital role in everyday life and contributes to mental and physical health and well-being [32]. Social support has been defined as the interactive process in which emotional concern, instrumental aid, information, and appraisal are obtained from one's social network. The most common types of support in online communities seem to be informational and emotional support [33], which can offer stability and help members manage uncertainty while preserving their autonomy and integrity in social interactions [34].

#### 3.1.4. Transition

*Emerging Adulthood.* During young adulthood (18–25 years) people develop their identity by exploring and experiencing different friendships and relationships and different educational and work possibilities over a longer period before they commit themselves to long-term choices. During these years, an independent exploration of possibilities in life becomes greater than at any other time [35], which can also lead to risky behaviors, such as unprotected sex and substance abuse, eventually leading to decreased health. Emerging adulthood also presents increasing stress, depression, and anxiety, probably a consequence of economic challenges, job dissatisfaction, and loneliness. Arnett [35] labels this period in life as emerging adulthood, characterized by less dependency on adults but still having not yet adapted to the responsibilities, traditionally normative for adulthood.

*Transition Theory.* Transitions have been described as critical situations and settings influencing the life course and life project. Changes in health status are also critical transitional situations. During transitions, people need to incorporate new skills and knowledge connected to a need for changed behaviors. Consequently, self-identity is challenged and requires reorientation or reconstruction of the sense of self. Transitional situations are thus connected to increased vulnerability [36, 37]. Meleis [34] has developed a “transition theory” related to health and illness which deals with this phenomenon.

### 3.2. Theory Use

In this section follow concise descriptions of how theories (Table 2) were utilized in each case, from the design process of the web support intervention to the evaluation of the web support prototype. This description is inspired by [14, 17, 21] and outlined in the four categories below.

#### 3.2.1. Theories Providing Fundamental Ideas behind the Cases

Preschool children with a long-term illness are at risk of psychosocial illness and poor compliance with treatment. Fundamental theories applied in Case 1 were learning [28] and health and well-being theories [28–32]. Fundamental ideas behind the project of young adults living close to mental illness came from theories of transition [33–35] as well as health and well-being theories included in the different learning sections of the websites [28–32] which encourage the young adults to learn, communicate, and feel community (Case 2). Among women with diabetes, the transition theory [34, 35] and the theories of health and well-being were central [28–32]. For these women, pregnancy and motherhood are important transitions in life and include increased vulnerability (Case 3). Lastly, for women who have undergone breast cancer surgery (Case 4), learning theories [25–27], and also the theory of [28], and health and well-being theories [28–32] were an important inspiration for the project's focus.

#### 3.2.2. Theories Informing the Organization of the Design Project in Each Case

A participatory design [22, 24] was used (Table 2) for all four projects. In the first case, the participating children expressed their opinions about pictures, and pediatric nurses, illustrators, and web designers took part in the process. For the second case of young adults, the working process took place in parallel with a test group online. Other participators included doctoral students, systems developers, and a communicator along with the researchers. For the third case of pregnant women and new mothers, an expert group of women with type 1 diabetes and experience of childbearing participated along with health care professionals of different specializations and the researchers. Finally, in the fourth case, women with breast cancer were involved in the design process via focus groups and in the decision-making during the design of the provided web-based program. Health care professionals and web designers also participated in the development along with the researchers.

#### 3.2.3. Theories Informing the Design of the Technology

In all of the cases, learning and health and well-being theories (Table 2) were central in the technology design. In Cases 1, 2, and 3, aspects of Community of Practice theory [25] were used in technology design, and in Cases 2, 3, and 4, Social Support Theory [30–32] was important in this respect as communities can mediate knowing, practice, create meaning, and promote identity development during vulnerable life phases and transitions.

#### 3.2.4. Theories Informing the Outcome Evaluation

Outcome measures to evaluate effects of the web-based support in the four cases were mainly related to the areas of health/well-being and/or learning. However, there are differences between cases if these two areas are used as primary or secondary outcome measures. The health measures include broader perspectives of health status, psychological well-being, self-esteem, and life satisfaction. Hypotheses that the actual web-support should increase health and/or well-being (in the broader perspective) were evaluated by using the following instruments: Case 1—content child index-CCI, I think I am-ITIA and for parents SOC-13; Case 2—WHO-5, SOC-13; Case 3—W-BQ12, SOC-13; and Case 4—FACT-B, SOC-13. Instruments used for evaluation of decreased negative dimensions of well-being were: Case 2—stress (PSS); Case 3—fear (HFS); and Case 4—anxiety and depression (HAD).

Utilized instruments also involved aspects of social learning, self-efficacy, self-determination, and empowerment, descending from social learning theories and social cognitive psychological theories. The following instruments were used to measure effect on these aspects: Case 1—parents manageability of the child's chronic illness (SOC-13); Case 2—self-efficacy (GSE) and perception of caring situation (COPE); Case 3—empowerment, manageability, and self-efficacy (SWE-DES-10, Swe-PAID); Case 4—participation in healthcare and information seeking skills (CHESS).

Notably, all theories identified as fundamental for the ideas behind the projects (cases) are not present as outcome measures. One example is in Case 3; the web-based support was developed to enable a secure transition into motherhood. This is not explicitly evaluated by using validated instruments. However, specifically contextual questions related to becoming a mother when having diabetes were used to capture perceptions of such issues.

### 3.3. To What Extent Are These Theories Applied in Line with the Ideal of Person-Centered Care? 

In this section we analyze if and how the theories used are connected to and compatible with the philosophy of person-centeredness. The analysis is described following the categorization of theories as used above.

#### 3.3.1. Design

PD is about being part of decisions affecting one's own situation or at least the technology that is being designed. It can be about taking part in the results of the design process, but it can also involve the user really influencing what is accomplished [24]. This is in line with PD as well as person-centeredness. In both, the person is involved as a potential user of technology who has the right to influence its design, maybe because (s)he belongs to a group with a chronic disease taking part in the design. However, it is up to the individual how much the aspect of being diagnosed with an illness influences his/her views and what (s)he does when taking part in a design project. In employing PD arrangements where professionals take part, the dimension of partnership is a logical aspect (c.f. Cases 1 and 4). However, as mentioned, use of PD in web-based support can also be a way of allowing the individual to influence the situation in the form of the technology that is designed. This may go in a direction that serves as a complement to the intention to create a therapeutic alliance between the person and the professionals. For example, the technology can serve to support closer interaction between patients and their social context, as in Cases 2 and 3, where the individuals were encouraged to share their experiences with peers in similar situations, or it could result in the design of more independent patient online groups [38].

#### 3.3.2. Learning

In person-centeredness, central parts of learning are the translation of learning into practice and how it can be meaningful for the person [8]. We argue that Communities of Practice and Sociocultural theory are compatible with person-centeredness in their focus on sharing experiences and knowledge from the individual's own point of view. This is thus in line with the holistic perspective and value that is put on the individuals' perspectives and resources in person-centeredness. Nevertheless, these theories also have a somewhat more explicit collective flavor in that their focus is on group interaction between individuals as a part of a learning process. This is not, we perceive, so marked in person-centeredness. Variation theory is less collective in the sense that it focuses on the person's understanding of the situation. Thus, if the learning processes are to be useful, they must respond to the capacities of the individual and thus have a person-centered approach.

Further, according to Sociocultural theory, technology supports learning but also influences the way learning takes place [27]. Consequently, as with person-centeredness, the individual, his/her mind and body, along with the technology affects and influences learning.

#### 3.3.3. Health and Well-Being

By its nature, the theory of salutogenesis emphasizes that the person is not his/her diagnoses or illness, as it stresses that every person has general resources which contribute to the endeavor of achieving better health [30]. Those aspects as well as the focus on the individual's own resources and capability are compatible with person-centeredness.

Another way of studying health is to assess a person's self-efficacy, which includes belief in own ability to manage difficult situations. Important aspects of self-efficacy are enactive mastery experience, which, if positive, will generate high self-efficacy in situations overall. Observation of how other people succeed in certain situations will also increase self-efficacy, especially if the role model has similar conditions. Verbal persuasion means that significant others have an impact through verbalizing the proposed capacity. Individuals thus supported will manage a situation and put more energy into doing the job, which in turn will make them succeed. The last part comprises physiological and affective states and the impact these have on the person in stressful situations. Like Communities of Practice and Sociocultural theory, social support theory also has a collective aspect or flavor. In other words, it may be that this support is obtained from peers rather than in an alliance between the person and the professional. As to the issue of taking part in decisions that affect one's own situation and illness, these theories are both compatible with this ideal of a supporting partnership.

#### 3.3.4. Transition

The movement from one state, condition, or place to another requires a person to incorporate new knowledge, to alter behavior, and to change the definition of self in the new social context [34]. Emerging adulthood and transition theories apply a holistic perspective, which is compatible with person-centeredness [34]. In other words, they take the individual's life situation, including contextual factors, into account. Transition is a central concept in nursing and transition theory has proved useful as a framework in the development of nursing projects with the aim of supporting healthy responses to transitions. Despite this, how the theory had influenced the only case [3] referring to it as a fundamental idea behind the project was not very explicit, as there was no acknowledgement of the specific conditions during transition to motherhood. However, some aspects of the technology design were informed by sorting information and threads in the social community according to the different phases of this transition.

In Case 2, the theory of emerging adulthood was used as a way of understanding how young adults managed their daily situations. However, when analyzing the processes of how they managed difficult situations, transition theory [34] was found to be more relevant than the theory of emerging adulthood. Knowledge of the young people's own retrospective reflections on their experiences and how they could have handled difficult situations differently [5] could be associated with transition theory [34], making this process more understandable. Taking an individual's self-reflection into account is central in PCC [10], thereby making transition theory more suitable for understanding the process than emerging adulthood theory. This is supported by [35], who states that transition theory is better suited for viewing the young person from a lifespan perspective.

## 4. Discussion

The theories used provided insight into the fundamental ideas behind the projects and informed the design of technology, the organization of design, and the outcome evaluation. Fundamental ideas behind the projects differed but encompassed health and well-being theories mixed with a variety of cases focused on transition or learning or both. In all cases, PD played an important role in the organization of the development work. PD can also be characterized as compatible with person-centeredness. The most common theories directly influencing the actual technology design, as well as outcome evaluation, were theories about learning and health and well-being. All the utilized theories were in many ways compatible with person-centeredness.

A notable exception was the relatively collective character of PD and some of the theories of learning (c.f. Communities of Practice). In person-centeredness, the individual is the center of attention, whereas utilization of a specific theory may more markedly promote group interaction among individuals. This is something that, we argue, complements the aim of strengthening the partnership with the providers. Nevertheless, partnership in the context of web-based support when used to assist daily life of chronic illness, as in the cases studied here, has different prerequisites compared to inpatient clinical practice. This has to be taken into account when evaluating compatibility with the component of partnership in PCC. On the other hand, shared power and responsibility can still be promoted in the web support, as illustrated. Lastly, all the utilized theories make assumptions that include a holistic perspective, which is in line with person-centeredness.

This study was pursued, at least partly, ex post facto. Consequently, ex post facto rationalization as well as exaggerations of theory use might exist. On the other hand, during the process, longitudinal data collection was carried out, as well as a profound and critical discussion of results. The cases also focused on the design of web-based support. An alternative might have been to collect more dispersed cases with different technology types to manage chronic illness [39, 40].

Further, other ideas about chronic illness include caregivers and family support and these factors were not explicitly addressed by this study, which emphasizes the person-centeredness. Although professional and family support is acknowledged to be of outmost importance, the main focus in the four projects was on strengthening of the person's capability and self-efficacy. This raises questions about an eventually conflicting aspect concerning the two concepts “person centeredness” and “family centeredness.” The latter is a concept central when supporting children with chronic illness as the family around the child is so essential and thus supports the need to really include the family as a whole.

Last but not least, the aim of this study was to provide a critical understanding of the roles of theories and their compatibility with a person-centered approach. Interventions like these can of course influence a person's way of managing daily life with the illness, that is, the behavior. There is an intention to make the person better equipped to manage a difficult situation through the provided support. This is in itself an important issue to study due to the proliferation of technology to be used by patients.

## 5. Limitations

We want to address two limitations in this study. The first being the potential for bias in our analysis of theory use due to the fact that the data was collected from authors' own cases. However, data was to a significant degree collected by the first author not being a full member of any of the four cases. The analysis was, on the other hand, pursued by all involved writers.

The other limitation we want to address is that data collection in two of our four interventions, Case 1 and 3, is not yet finalized. Thus outcomes are not available for analysis of in what ways the developed support had been of help in managing chronic illness. Due to this fact, we are not able to draw any conclusions of the relevance of included theories in relation to outcome measures.

## 6. Conclusions

The focus of theory used in the design and evaluation of web-based support can be on design, learning, and health and well-being as well as transition (Table 2). In this study we discussed four different cases of theory use in contrast to “theoretical discussion of theories” [15, 20, 21] or single qualitative case studies [14, 15, 18]. It is important to note that theories can be viewed as a complement to user views [11, 13] as well as to the more implicit ideas and values of researchers [41]. Our results therefore illustrate the multifaceted ways in which theories can provide visible and traceable grounds for web-based support. Theories are equally important, irrespective of whether the caring situation includes web-based technologies or is face to face. In both situations, we need to be explicit about how to meet the needs of people with chronic illness.

The message of this paper is that theory awareness as a result of current and ex post facto reflections will strengthen the quality of research. This greater awareness also serves to improve knowledge of whether or not the utilized theories are compatible with person-centeredness.

## Figures and Tables

**Table 1:** Characteristics of the four cases of web-based support included∗.

	Case 1	Case 2	Case 3	Case 4
Target group	Preschool children (aged 4–6) with bladder dysfunction and urogenital malformation.	Young adults (aged 16–25) living with mental illness.	Women with type 1 diabetes who are pregnant or in early motherhood (up to infancy of 6 months).	Women who have undergone surgery for breast cancer.

Components of web support	Web support with specially developed themes of pictures and stories. Communication between children and a “web teacher” using Skype.	Web support for providing information, learning, and self-care as well as peer and professional support.	Web support for providing information, self-management tool for documentation, and peer support.	CD disk and web support with information and expert lectures on different topics (medical facts and social and psychological aspects)

Design of trial	Consecutive selection with matched controls	Randomized, controlled trial	Randomized, controlled trial	Randomized, controlled trial

^*^A similar table of these basic aspects was published in [22].

**Table 2:** Theories used in the development of web-based support.

Type of theory	Case 1 (Barnweb)	Case 2 (PS Young support)	Case 3 (MODIAB-Web)	Case 4 (Siri-B)
Design				
Participatory design	X	X	X	X
Learning				
Communities of Practice	X	X	X	X
Sociocultural perspective				X
Variation theory	X			
Health and well-being				
Salutogenic theory	X	X	X	X
Social support		X	X	X
Transitions				
Emerging adulthood		X		
Transition theory			X	
